# Supplementary material for: Light-inducible antimiR-92a as a therapeutic strategy to promote skin repair in healing-impaired diabetic mice
Source: Nat Commun. 2017 May 2;8:15162. doi: 10.1038/ncomms15162 (PMC5418571; doi:10.1038/ncomms15162)
Supplement: Supplementary Information — Supplementary figures. [file ncomms15162-s1.pdf]

## Supplementary Figure 1

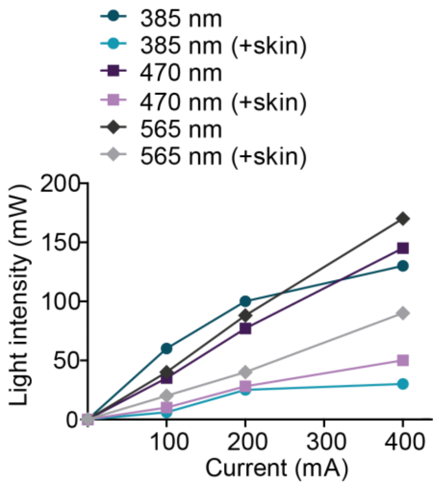


**Supplementary Figure 1**: Measurement of light intensity with or without murine skin tissue as a barrier by using different wavelengths and currents as indicated. The experiment was performed once for each condition with one skin explant isolated from one male Bl/6 mouse.

Supplementary Figure 2

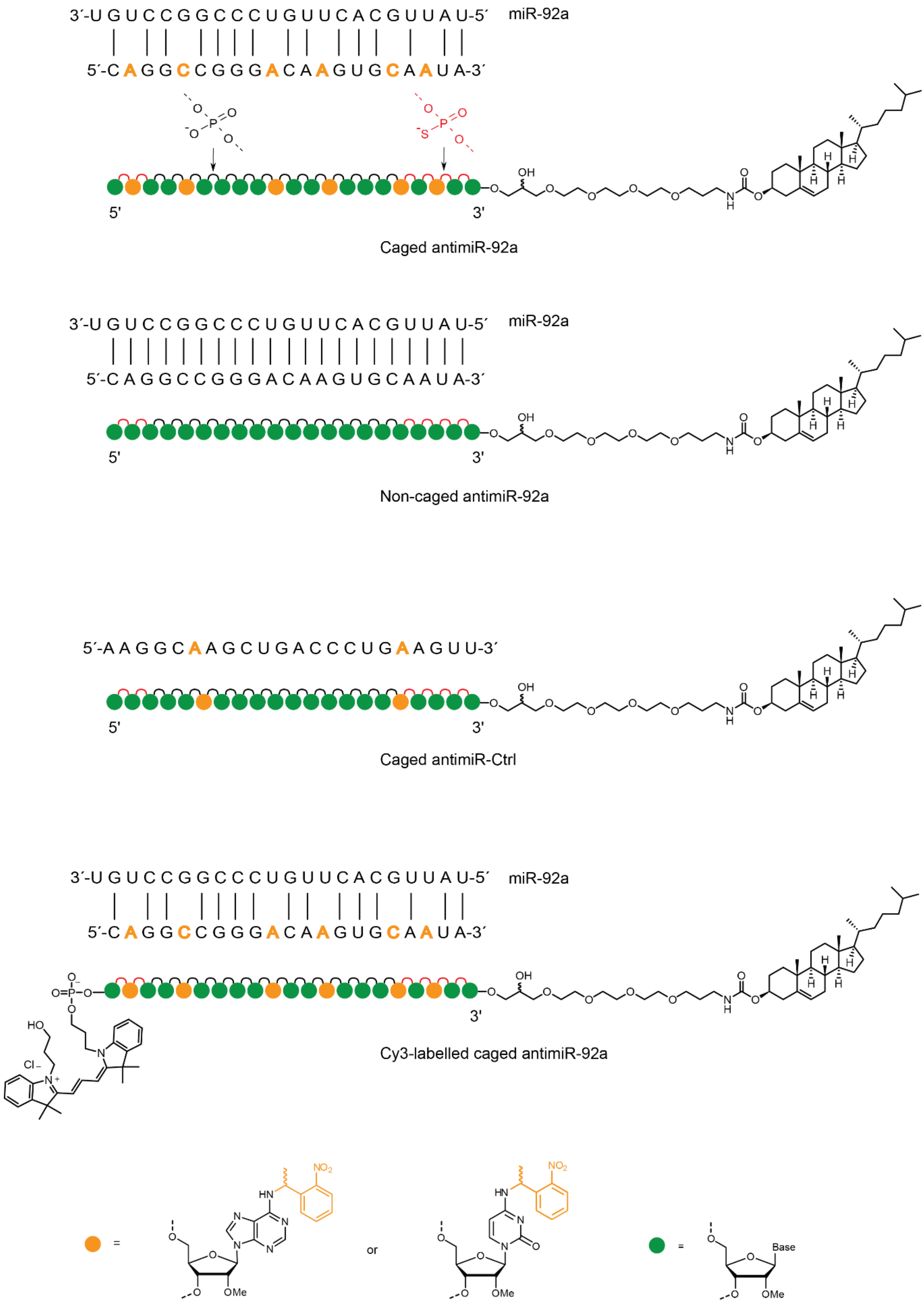


**Supplementary Figure 2:** Schematic representation of the chemical structure of caged antimiRs.

Supplementary Figure 3

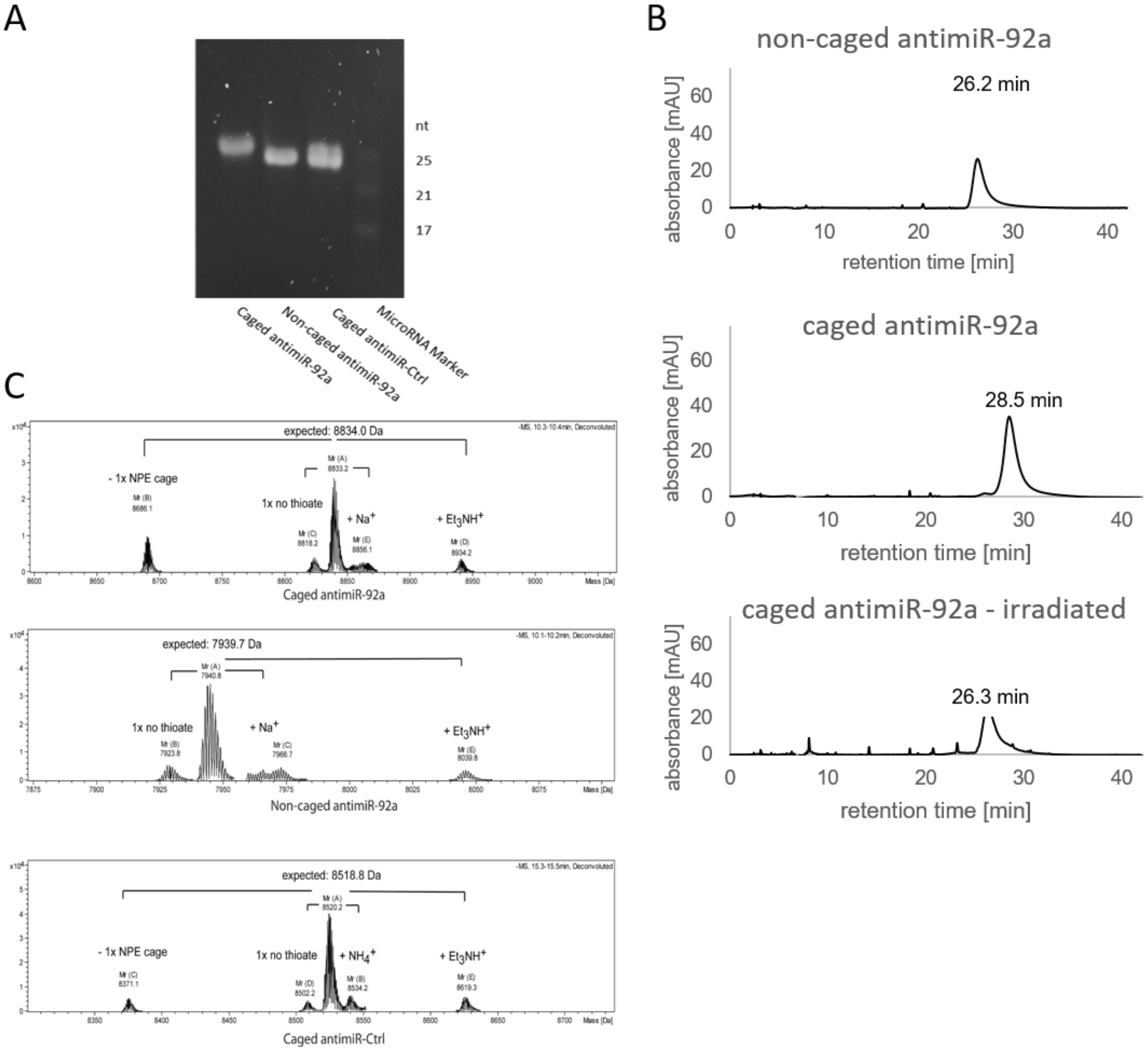


**Supplementary Figure 3. (A)** Analytical denaturing PAGE (18%) of light-inducible antimiRs and microRNA Marker (NEB), which were stained with SYBR Gold (Thermo Fisher Scientific) and photographed on a transilluminator. **(B)** The purity of the non-caged antimiR-92a (top panel), caged antimiR-92a (mid panel) and the release of the active antimiR after irradiation (bottom panel) were demonstrated via analytical HPLC on an Agilent 1200 series system with a Multokrom 100-5 C18 column (250 x 4.6mm) using a dual buffer system containing 0.1 M triethylammoniumacetate in water pH 7 (buffer A) and acetonitrile (buffer B) and a two-step linear gradient (Step 1: 5-20% B in 4.1 minutes, Step 2: 20-90% B in 34.9 minutes). Each sample contained 500 pmol of oligonucleotide in PBS buffer. Irradiation was performed with a custom-built 365 nm UV-LED device (I = 0.5 A, P = 250 mW). The caged sample was irradiated in a glass vial for 10 minutes. The spectrum of all was background-subtracted against a PBS blank injection and the detection wavelength was 254 nm. **(C)** Top panel, deconvoluted ESI-LC/MS spectrum of caged antimiR-92a. The calculated mass of the full-length

product is 8834.0 Da. Mid panel, deconvoluted ESI-LC/MS spectrum of non-caged antimiR-92a. The calculated mass of the full-length product is 7939.7 Da. Bottom panel, deconvoluted ESI-LC/MS spectrum of caged antimiR-Ctrl. The calculated mass of the full-length product is 8518.8 Da. Bruker mirOTOF-Q II, column: MultoKrom 128 mm x 2 mm, 5% -> 100% MeCN/5 mM $Et_3NHOAc$ pH 7.0 in 11 min, 0.3 mL/min). The spectra show salt adducts and a small fraction of species with one less caging group or one less phosphorothioate linkage and a phosphodiester linkage instead.

Supplementary Figure 4

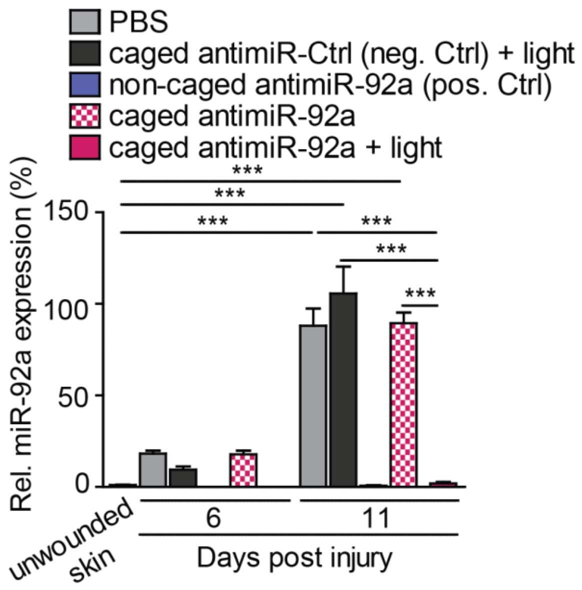


**Supplementary Figure 4: Repetitive caged antimiR-92a injection downregulates miR-92a levels in cutaneous wounds.** Quantification of miR-92a levels via qPCR 6 and 11 days post wounding. Mice were treated immediately upon wounding as well as on day 4 and 7 post injury with antimiRs, with or without light activation as indicated. n = 4-14 wounds/group on 3-7 different mice. Data are expressed as means ± SEM. Significance of difference was analyzed using ANOVA one-way test analysis with Newman-Keuls Multiple Comparison Test.***p<0.001.

## Supplementary Figure 5

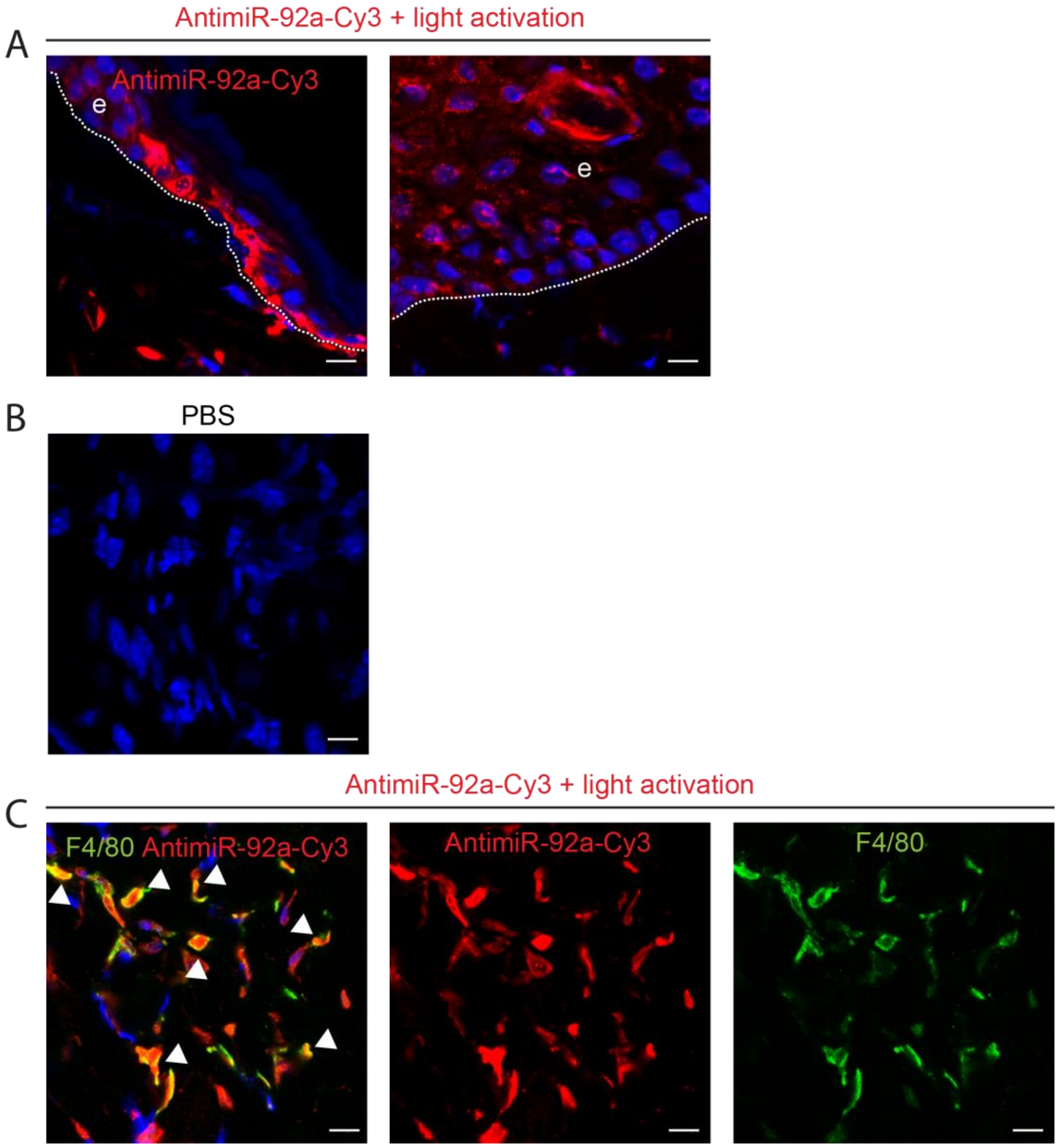


**Supplementary Figure 5: Distribution of Cy3-labelled caged antimiR-92a upon i.d. injection. (A)** Two representative overview images of Cy3-labelled caged antimiR-92a treated wounds with light activation 6 days post injury. Dashed line marks basement membrane; e, hyperproliferative epithelium. **(B)** PBS treated wound were used as negative controls. **(C)** Representative images of Cy3-labelled caged antimiR-92a treated wounds and counterstaining with F4/80 to highlight macrophages. Arrowheads point to F4/80/Cy3 double positive cells. Left panel, merge image; mid panel, Cy3 image; right panel, F4/80 single staining. Nuclei are stained with Dapi. Bar graph, 10 µm. n=3 animals.

Supplementary Figure 6

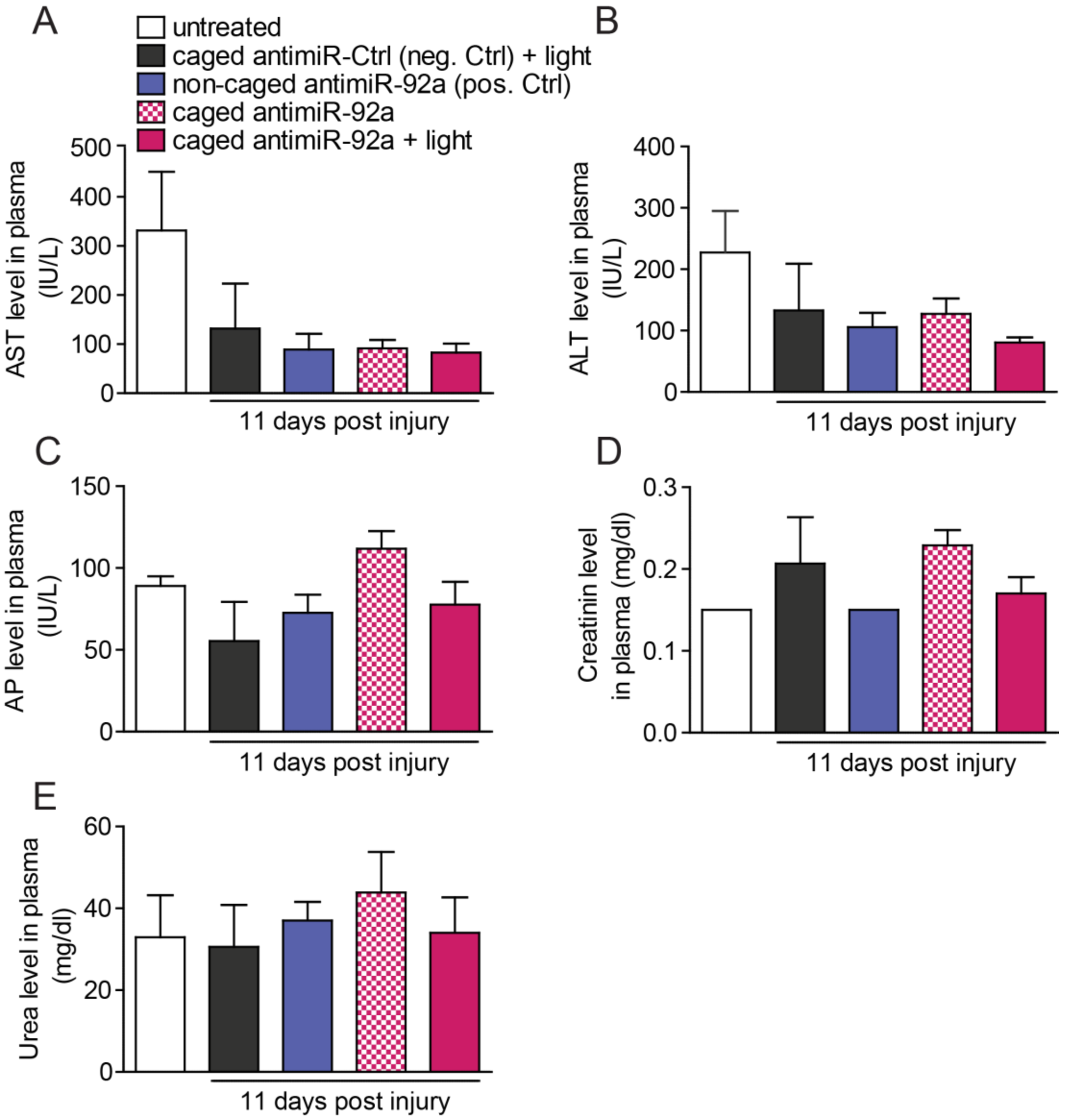


**Supplementary Figure 6: Caged antimiR-92a injection shows no toxic side effects in excretory organs.** Quantification of **(A)** AST (aspartate aminotransferase), **(B)** ALT (alanine aminotransferase); **(C)** AP (alkaline phosphatase) , **(D)** creatinine and **(E)** urea plasma levels in untreated db/db mice as well as in wounded db/db mice treated three times with caged antimiRs as indicated. Plasma was isolated 11 days post wounding. n = 3-4 mice/group. Data are expressed as means ± SEM. Significance of difference was analyzed using ANOVA one-way test analysis with Newman-Keuls Multiple Comparison Test.

Supplementary Figure 7

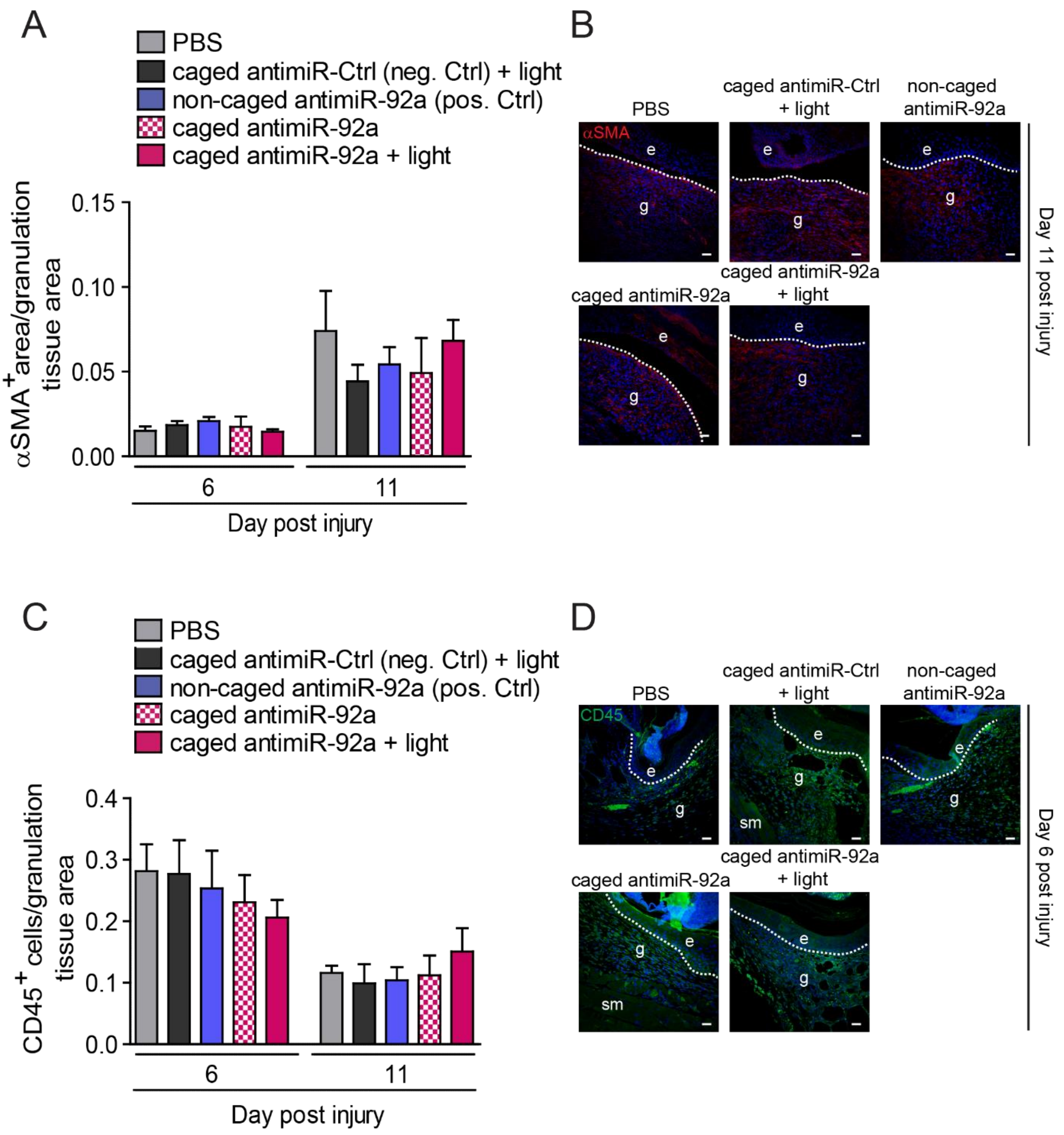


**Supplementary Figure 7: miR-92a inhibition does not influence myofibroblast differentiation and inflammation. (A)** Morphometric quantification of immunohistochemically stained αSMA+ area and **(B)** representative images of wound sections 11 days post injury stained for αSMA. **(C)** Quantification of CD45+ stained cells within the granulation tissue upon caged antimiR treatment as indicated and **(D)** representative images of wound sections 6 days post injury stained for CD45. Different groups as indicated. n=4-14 wounds/group on 2-7 different mice. Bar graph, 50 µm; e, hyperproliferative epithelium; g, granulation tissue; sm, skeletal muscle. Data are expressed as means ±SEM. Significance of difference was analyzed using ANOVA one-way test analysis with Newman-Keuls Multiple Comparison Test.

Supplementary Figure 8

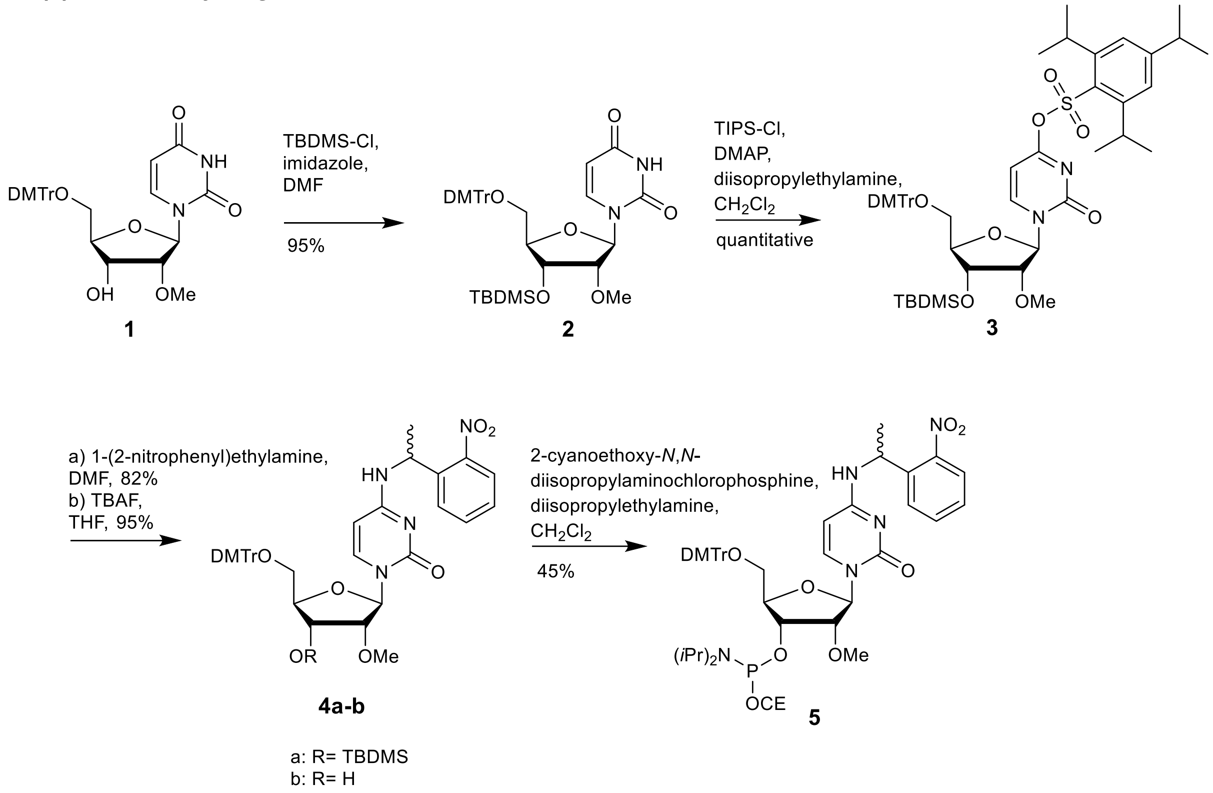


**Supplementary Figure 8.** Synthesis of 2´-OMe cytidine phosphoramidite.

Supplementary Figure 9

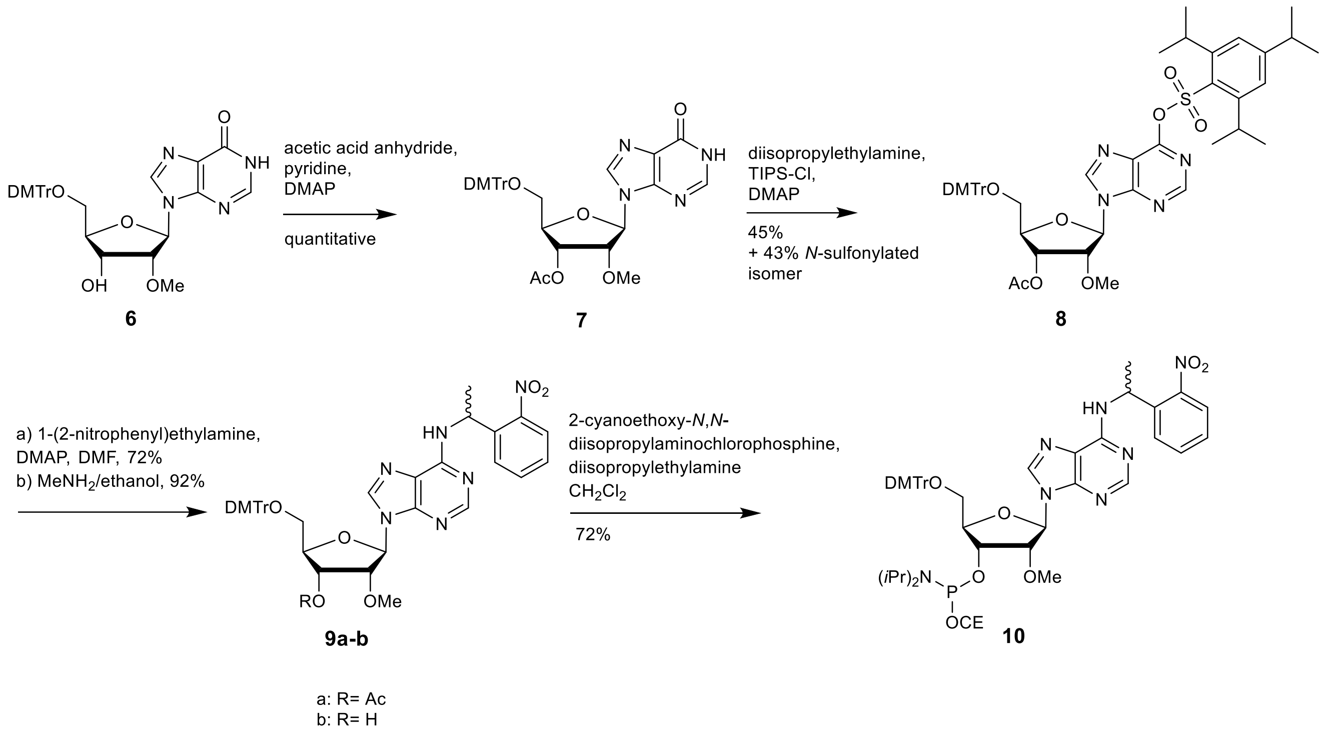


**Supplementary Figure 9.** Synthesis of 2´-OMe adenosine phosphoramidite.
